# Medical trigger teams for severely ill medical patients in the Emergency Department

**DOI:** 10.1186/1757-7241-23-S1-A51

**Published:** 2015-07-16

**Authors:** Stefan Posth, Mette Worsøe, Annmarie T Lassen

**Affiliations:** 1 Department of Emergency Medicine, OUH Odense University Hospital, Odense, Denmark

## Background

Medical patients who arrive severely ill at the Emergency Department (ED) need to be diagnosed and treated immediately, as prognosis decreases rapidly with time spent before correct treatment is given for most of these patients.

At Odense University Hospital, patients arriving at the ED with either a pulse rate >140, systolic blood pressure <80 mmHg, Glasgow Coma Scale <9, respiratory frequency >35 or <8 or a saturation <80% are met by the medical trigger team. Patients with suspected apoplexy, STEMI or orthopedic multitrauma are met by other specialized teams.

## Objectives

To describe frequency, patient characteristics, and 7 day mortality for adult patients (>18 years) managed by medical trigger teams, thrombolysis, and orthopedic trauma teams.

## Methodology

All patients who were met by a medical trigger team, a thrombolysis, or an orthopedic trauma team at arrival to the ED, Odense University Hospital between April 1st 2012 and May 31th 2013 were included.

## Results

A total of 884 patients were met by the medical trigger team, 306 by the thrombolysis and 377 by the orthopedic multitrauma team. The median (range) number of team activations per day was 2 (0-9), 0(0-6), and 1 (0-6). Median age (25-75%) was 70 (56-80), 67 (54-76), and 42 years (26-59), 54% (95% CI 50%-57%), 53% (95% CI 48-59%), and 73% (95% CI 68-78%) were male, 7 day mortality was 18% (95% CI 16-21%), 9% (95% CI 6-13%) and 7% (95% CI 5-11%).

## Conclusion

Medical trigger teams were used median twice a day. Their patients have a higher mortality than patients met by the thrombolysis or orthopedic multitrauma teams.

